# Encapsulation into Stealth Liposomes Enhances the Antitumor Action of Recombinant *Cratylia mollis* Lectin Expressed in *Escherichia coli*

**DOI:** 10.3389/fmicb.2016.01355

**Published:** 2016-09-16

**Authors:** Cássia R. A. da Cunha, Luís C. N. da Silva, Fábio J. F. Almeida, Milena S. Ferraz, Nathalia Varejão, Marina F. de Souza Cartaxo, Rita de Cássia M. de Miranda, Francisco C. A. de Aguiar, Noemia P. da Silva Santos, Luana C. B. B. Coelho, Nereide S. Santos-Magalhães, Maria T. dos Santos Correia

**Affiliations:** ^1^Laboratório de Bioquímica de Proteínas, Departamento de Bioquímica, Universidade Federal de PernambucoRecife, Brazil; ^2^Programa de Pós-Graduação em Biologia Parasitária, Universidade CeumaSão Luís, Brazil; ^3^Laboratório de Imunopatologia Keizo-Asami, Universidade Federal de PernambucoRecife, Brazil; ^4^Laboratório de Agregação de Proteínas e Amiloidoses, Instituto de Bioquímica Médica, Universidade Federal do Rio de JaneiroRio de Janeiro, Brazil; ^5^Programa de Pós-Graduação em Meio Ambiente, Universidade CeumaSão Luís, Brazil; ^6^Centro Acadêmico de Vitória, Universidade Federal de PernambucoVitória de Santo Antão, Brazil

**Keywords:** lectins, heterologous expression, cancer, immunomodulation, pharmaceutical preparations

## Abstract

This study evaluated the *in vivo* antitumor potential of the recombinant lectin from seeds of *Cratylia mollis* (rCramoll) expressed in *Escherichia coli*, free or encapsulated in stealth liposomes, using mice transplanted with sarcoma 180. rCramoll-loaded stealth liposomes (rCramoll-lipo) were formulated by hydration of the lipid film followed by cycles of freezing and thawing, and about 60% of rCramoll was encapsulated. This novel preparation showed particle size, polydispersity index, and pH suitable for the evaluation of antitumor activity *in vivo*. Tumor growth inhibition rates were 59% for rCramoll and 75% for rCramoll-lipo. Histopathological analysis of the experimental groups showed that both free and encapsulated lectin caused no changes in the kidneys of animals. Hematological analysis revealed that treatment with rCramoll-lipo significantly increased leukocyte concentration when compared with the untreated and rCramoll group. In conclusion, the encapsulation of rCramoll in stealth liposomes improves its antitumor activity without substantial toxicity; this approach was more successful than the previous results reported for pCramoll loaded into conventional liposomes. At this point, a crucial difference between the antitumor action of free and encapsulated rCramoll was found along with their effects on immune cells. Further investigations are required to elucidate the mechanism(s) of the antitumor effect induced by rCramoll.

## Introduction

Cancer is a set of more than 100 pathologies with a related feature: genetic mutations result in an uncontrollable growth of cells that can invade tissues and organs (Ryan and Bernstein, 2012). The World Health Organization estimates that 17 million people will die of cancer in 2030. The battle against a disease of this magnitude comprises: (i) finding the right target for tumor inhibition; (ii) discovering drugs that effectively treat this disease; and (iii) developing the most successful form to transport this drug (Danhier et al., 2010).

Various plant-derived compounds are potential candidates for treatment of cancer, such as lectins, a very large class of proteins, which bind specifically and reversibly to carbohydrate residues (Da Silva and Correia, 2014). For years, these proteins have been used as a tool to differentiate benign from malignant tumors, by assessing alterations in the glycosylation pattern between normal and cancer cells (Ghazarian et al., 2011). In addition to the diagnostic potential, some lectins have antitumor activity, mainly, targeting apoptosis pathways (Liu et al., 2010; Fu et al., 2011). One of these antineoplastic lectins is Cramoll 1,4 (or pCramoll) extracted from the seeds of *Cratylia mollis*, which showed an antitumor action *in vivo* against sarcoma 180 (Andrade et al., 2004). In order to avoid seasonal interference and provide purer protein preparations, a recombinant lectin (rCramoll) was heterologously expressed in *Escherichia coli* (Varejão et al., 2010). This new lectin has the same ability to agglutinate erythrocytes, monosaccharide specificity, and share several other biophysical properties of native protein, however, rCramoll seems to be more stable in denaturing situations (Varejão et al., 2010, 2011).

Despite the anticancer potential of lectins, there are some obstacles in using these macromolecules for this purpose, such as the possible degradation by biological fluids, action on unwanted targets, and the need for frequent doses to maintain a therapeutic level (Cleland et al., 2001). These difficulties can be overcome by the development of nanosystems, and one good example is liposomes, vesicles consisting of one or more lipid bilayers which may carry both hydrophilic, and lipophilic drugs (Torchilin, 2011). Specifically, the stealth liposomes have been shown as an effective tool for protein encapsulation due to their higher circulation time and passive accumulation in tumor tissues. All these beneficial effects of stealth liposomes are related to the enhanced permeability and retention (EPR) effect, whereby macromolecules can selectively and specifically pass through the blood vessels that permeate the tumor and accumulate only in these malignant tissues (Fang et al., 2011). The improved permeability and retention are results of the characteristic presence of leaky vascular system and disorganized lymphatic system in malignant tumors (Sur et al., 2014). In this context, the present work aimed to evaluate the *in vivo* antitumor activity of rCramoll and rCramoll-loaded stealth liposomes.

## Materials and methods

### Materials

Soya phosphatidylcholine (Epikuron® 200) and 1,2-distearoyl-sn-glycero-3-phosphoethanolamine-N-methoxy-poly(ethylene glycol 2000; DSPE-PEG2000) were obtained from Lipoid (Dusseldorf, Germany). Trehalose and Total Protein Peterson-Lowry Kit were purchased from Sigma-Aldrich (St. Louis, USA). The dialysis membrane (*cut off* 300 kDa) was purchased from Spectrum Labs (USA). All other reagents used were of analytical grade and purchased from Merck (Darmstadt, Germany).

### rCramoll expression and experimental factorial design

rCramoll was obtained by heterologous expression using the expression vector pET-28a-Cramoll and the strain *E. coli* Rosetta (DE3), followed by purification using Sephadex G-75 column as described by Varejão et al. (2010). A 2^4^ full factorial design was used to study the effects of some factors on the expression level of soluble rCramoll: Cell density at induction measured as optical density at 600 nm (OD_600_; 0.5 or 4.0), isopropyl-β-D-thiogalactoside (IPTG) concentration (0.05 or 1 mM), time of induction (2 or 22 h), and temperature during the induction (37 or 15°C). The combinations resulted in eight different expression experiments, which were performed in triplicate. Three repetitions at the center point level (OD_600_: 2.25; 0.55 mM of IPTG, 26°C and 11.5 h) were also performed to determine if there is a non-linear relationship between the variables and the responses. After the expression experiments, the *E. coli* cells were harvested by centrifugation and disrupted by sonication on ice (Varejão et al., 2010). Soluble and insoluble fractions were separated by centrifugation and the presence of recombinant Cramoll was analyzed by denaturing SDS-PAGE electrophoresis. The protein concentration was quantified on scanned gels using the Quantity One software (Bio-Rad). Purified rCramoll was applied on gels at 5 μM as a standard for the quantification.

### Hemagglutination assays

The evaluation of hemagglutination activity (HA) was performed using rabbit erythrocytes, following the protocol proposed by Correia and Coelho (1995). Briefly, fresh erythrocytes from rabbit were obtained from auricular vein, fixed in glutaraldehyde and an erythrocytes suspension (2.5% v/v) was prepared in NaCl 0.15 M. In parallel, each lectin was submitted to a two-fold dilution (final volume of 50 μL) in NaCl 0.15 M using a microdilution plate (V-bottom). Each well received 50 μL of a rabbit erythrocyte suspension. After 45 min at room temperature, the highest dilution with visible hemagglutination was recorded. The HA was expressed in hemagglutination units (HU) which is defined as the inverse of the highest dilution displaying visible hemagglutination.

### Evaluation of functional stability of rCramoll after ultrasound, mechanical agitation and cycles of freezing and thawing

Samples of rCramoll (150 μg/mL) were submitted to ultrasound (200 W, 40 Hz at 4°C for 100, 250 and 300 s) and mechanical agitation (150 rpm at 37°C for 24 or 48 h). For the stability test after cycles of freezing and thawing, rCramoll (1 mL at 150 μg/mL) in phosphate buffer (pH 7.4) was subjected up to 6 cycles of freezing in liquid nitrogen (–196°C) and thawing in a water bath (35°C). After these procedures, the hemagglutination activity (HA) was evaluated. All these studies were performed in triplicate.

### Liposome preparation

The liposomes were obtained by freeze-thaw technique (Pick, 1981). Briefly, lipids (phosphatidylcholine: Cholesterol: DSPE-PEG2000) at the desired ratio (7.5:2:0.5) were dissolved in a chloroform/methanol solution (3:1). Organic solvents were removed under reduced pressure (37 ± 1°C at 80 rpm) for formation of the lipid film. After, the film was hydrated with a solution of lectin (700 μg/mL) in pH 7.4 phosphate buffer (Andrade et al., 2004), and subjected to two cycles of freezing and thawing for the formation of MLV's (large multilamellar liposomes vesicles), and finally sonicated for 25 s (200 W, 40 Hz and 4°C) to obtain small unilamellar vesicles (SUV).

### Optimization and stability assessment of liposome formulations

The optimization of particle size and polydispersity index (PI) of liposome formulations were performed using different sonication times (0, 10, 25, 50, and 100 s). The mean particle diameter and polydispersity index of liposomes were determined by photon correlation spectroscopy (PCS) using a laser particle size analyzer Delsa™ Nano S (Beckman Coulter, UK). The surface charge (zeta potential) of the vesicles was determined using a Zetatrac Legacy (Microtrac®, USA). Furthermore, the long-term stability was evaluated by pH, particle size and polydispersity index measurements after 7, 15, 30, 45, 60, and 90 days. For all analysis, liposome samples (*n* = 3) were diluted in ultrapurified water (25°C) for particle counting. The results obtained are shown as mean ± standard deviation (SD).

### Encapsulation efficiency

To assess the rCramoll encapsulation effectiveness, an aliquot of liposome (150 μL) was diluted in 50 μL of chloroform/methanol solution (3:1) and sonicated for 7 min. After, the volume was adjusted to 1 mL with Milli-Q water and the protein content was measured by Peterson-Lowry Kit, following the manufacturer's instructions.

On the other hand, the measurement of unencapsulated rCramoll was obtained using two approaches. The first consisted in an ultracentrifugation of liposome formulation (256,860 × g for 30 min; 4°C, Beckman Optima LE-80K, rotor 70 ti), followed by protein quantification in the supernatant. The second methodology employed a dialysis separation using 300 kDa cut-off membranes for 12 h with changes every 2 h, and finally, the protein quantification was performed as described above.

### Antitumor activity

The *in vivo* antitumor activity was evaluated using Sarcoma-180. Four groups (*n* = 7) were randomized into positive and negative controls, rCramoll and rCramoll-loaded liposomes treated groups. Ascitic tumor cells (5.0 × 10^6^ cells/mL) were inoculated subcutaneously (except for negative group) in the right axillary region of male Swiss mice (40 g body average weight; 40–60 days old). The treatment was started 24 h after tumor inoculation and lasted for 7 consecutive days, through one daily injection (7 mg/Kg/day) of rCramoll, rCramoll-loaded liposome or control group untreated (saline solution) intraperitoneally administered (Andrade et al., 2004). After treatment period, the animals were euthanized with Urethame® (1.25 g/kg body weight) and sacrificed by cardiac puncture. The antitumor activity was calculated using this formula:

$$\begin{array}{l} \text{Tumor inhibition }\left(\text{TI\%}\right):\text{ TI\%}=\left[\left(\text{C}-\text{T}\right)/\text{C}\right]\;\times \;100, \end{array}$$

where C is the mean tumor weight of the control group and T is the mean tumor weight of treated groups.

Experiments involving animals were performed 25 ± 2°C on 12 h light/12 h dark cycles with free access to water and food. All animal experiments were performed as proposed by ethical standards of Universidade Federal de Pernambuco and were approved by its ethics committee (CEUA/UFPE; protocol 23076.023165/2012-13).

### Histopathological analysis

At the end of the treatment phase, the tumor and some organs (liver, spleen, kidneys) were fixed in 10% neutral buffered-formalin solutions (24 h) and subsequently immersed in paraffin. Sections (3–5 μm) were prepared and slides were mounted for histological analysis. Slides were stained with hematoxylin and eosin and observed under a light microscope (Nikon-E200, Nikon Instruments). The mitotic index (MI) was determined evaluating mitosis number per field in each experimental group. Control animals were considered as 100%. Photomicrographs were evaluated using the ImageJ (version 1:44; Research Services Branch, USA).

### Hematological and biochemistry analysis

Blood samples were collected in MiniCollect® K3EDTA tubes (Greiner Bio-One, Austria) for hematological analysis, which included the determination of hemoglobin concentration (Hb), hematocrit (Ht), mean corpuscular volume (MCV), mean corpuscular hemoglobin (MCH), mean corpuscular hemoglobin concentration (MCHC), white blood cells (WBC), and red blood cells (RBC; SYSMEX XT-4000*i*™ automated hematology analyzer, Sysmex America, Inc.). The plasma levels of aspartate aminotransferase (AST), alanine aminotransferase (ALT), urea and glucose were determined using COBAS® 6000 automated analyzer (Roche Diagnostics, England). Additionally, the glutathione peroxidase (GPX) activity was estimated using Ransel Glutathione Peroxidase kit (Randox Laboratories, United Kingdom), according to the manufacturer's instructions.

### Statistical analysis

Data were analyzed by one-way analysis of variance (ANOVA) and Tukey test to determine the statistical significance using GraphPad Prism (GraphPad Software, Inc., San Diego, CA, versão 6.03). A *p*-value of < 0.05 was considered to be statistically significant. All data are represented as mean value ± standard deviation.

## Results

### rCramoll expression and experimental factorial design

**Figure A1:**
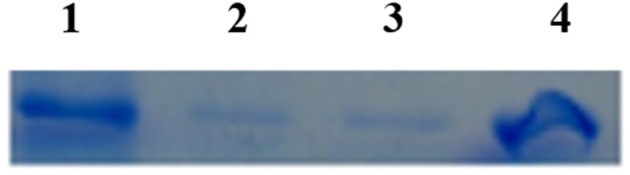
**Analysis of the soluble recombinant rCramoll produced by ***E. coli*** at the optimal culture conditions (Cell density of 0.5; IPTG at 0.1 mM; induction at 15°C and for 21 h**. 1-Total production of rCramoll in *E. coli*; 2-rCramoll in *E. coli* soluble fraction; 3-rCramoll in *E. coli* insoluble fraction; 4-Previously purified rCramoll (5 μM) used as a standard for the quantification.

An important challenge for high level expression of heterologous proteins in *E. coli* is the formation of inclusion bodies (Varejão et al., 2010). We investigated through experimental factorial design the effects of four culture conditions factors (such as induction cell density, IPTG concentration, temperature and time of induction) known to influence protein expression in *E. coli*. The results showed that the highest ratios of soluble/insoluble rCramoll were obtained in assays 1 and 3. In these two assays, the induction occurred at the lowest temperature of induction (15°C) and lowest cell density (0.5). A linear relationship between the tested variables and responses was found as the ratios observed for the center point level assays were within the value range obtained for each responses of the eight independent assays (Table 1 and Figure A1).

**Table 1:** **Experimental design matrix and response for expression of soluble rCramoll in ***Escherichia coli*****.

**Assay**	**Cell density (OD_600_)**	**IPTG Concentration (mM)**	**Temperature of induction (°C)**	**Time of induction (h)**	**Ratio of soluble/insoluble rCramoll**
1	0.5	0.1	15	21	1.27
2	4.0	0.1	15	2	0
3	0.5	1.0	15	2	0.83
4	4.0	1.0	15	21	0
5	0.5	0.1	37	2	0
6	4.0	0.1	37	21	0
7	0.5	1.0	37	21	0
8	4.0	1.0	37	2	0
9 (C)	2.25	0.55	26	11.5	0.41
10 (C)	2.25	0.55	26	11.5	0.34
11 (C)	2.25	0.55	26	11.5	0.20

### Pre-formulation assessment of rCramoll functional stability

All parameters of liposome preparation must ensure high drug encapsulation efficiency and preservation of biological activity of target protein (Walde and Ichikawa, 2001). In this context, evaluations of the influence of ultrasound, mechanical agitation, and freezing-thawing cycles on HA levels of rCramoll were performed after the formulation of liposomes. Samples submitted to ultrasound until 250 s did not show changes in their HA as compared to untreated rCramoll (256^−1^). Similarly, the HA of this protein was only altered after 48 h of mechanical agitation. In addition, the HA remained unchanged after 2 cycles of freezing-thawing (Table 2).

**Table 2:** **Effects of ultrasound, mechanical agitation, and cycles of freezing and thawing on hemagglutination activity of rCramoll**.

**Parameter**		**Hemagglutination activity (HA/50 μL)^a^**
Time of mechanical agitation (h)	0	256^−1^
	24	256^−1^
	48	128^−1^
Time of ultrasound (s)	0	256^−1^
	100	256^−1^
	250	256^−1^
	300	128^−1^
Cycles of freezing and thawing	0	128^−1^
	1	128^−1^
	2	128^−1^
	3	32^−1^
	4	32^−1^
	5	32^−1^
	6	32^−1^

a *The value is expressed as hemagglutination titer, defined as the reciprocal of the highest dilution exhibiting visible hemagglutination*.

### Characterization of rCramoll-loaded stealth liposomes

Based on the pre-formulation results, rCramoll-lipo were prepared using two freezing-thawing cycles and then submitted to different times of ultrasound (0–100 s). Taking into account that liposomes with size less than 200 nm have been described as appropriate for antitumor therapy (Allen and Cullis, 2013), the best formulation with a homogeneous size distribution was obtained after 25 s of sonication, showing the size of 168.0 ± 0.7 nm and PI of 0.265 ± 0.025, zeta potential of −31.9 ± 0.1 mV and pH 7.4 (Table 3).

**Table 3:** **Effects of ultrasound on the stability of rCramoll in stealth liposomes**.

	**Ultrasound time (s)**				
	**0**	**10**	**25**	**50**	**100**
Particle size (nm)	443.6 ± 34.7	230.7 ± 3.9	168.0 ± 0.7	106.1 ± 4.2	88.3 ± 0.4
Polydispersity index	0.463	0.299	0.265	0.226	0.219

Liposomes showed a long-term stability, after 90 days they continued in appropriate size (170.1 ± 2.0 nm), PI of 0.320 and pH of 7.3. The encapsulation efficiency of rCramoll evaluated by dialysis and ultracentrifugation were 57.3 ± 0.3% and 59.3 ± 1.5%, respectively. No statistical differences were observed between them (*p* < 0.05) (Table 4).

**Table 4:** **The encapsulation efficiency of rCramoll in stealth liposomes**.

**Method**	**Teor (%)**	**Encapsulation efficiency (%)**
Dialysis	95.82 ± 0.79	57.3 ± 0.3
Ultracentrifugation	95.82 ± 0.79	59.3 ± 1.5

### Antitumor activity of rCramoll and rcramoll-loaded stealth liposomes

**Figure 1:**
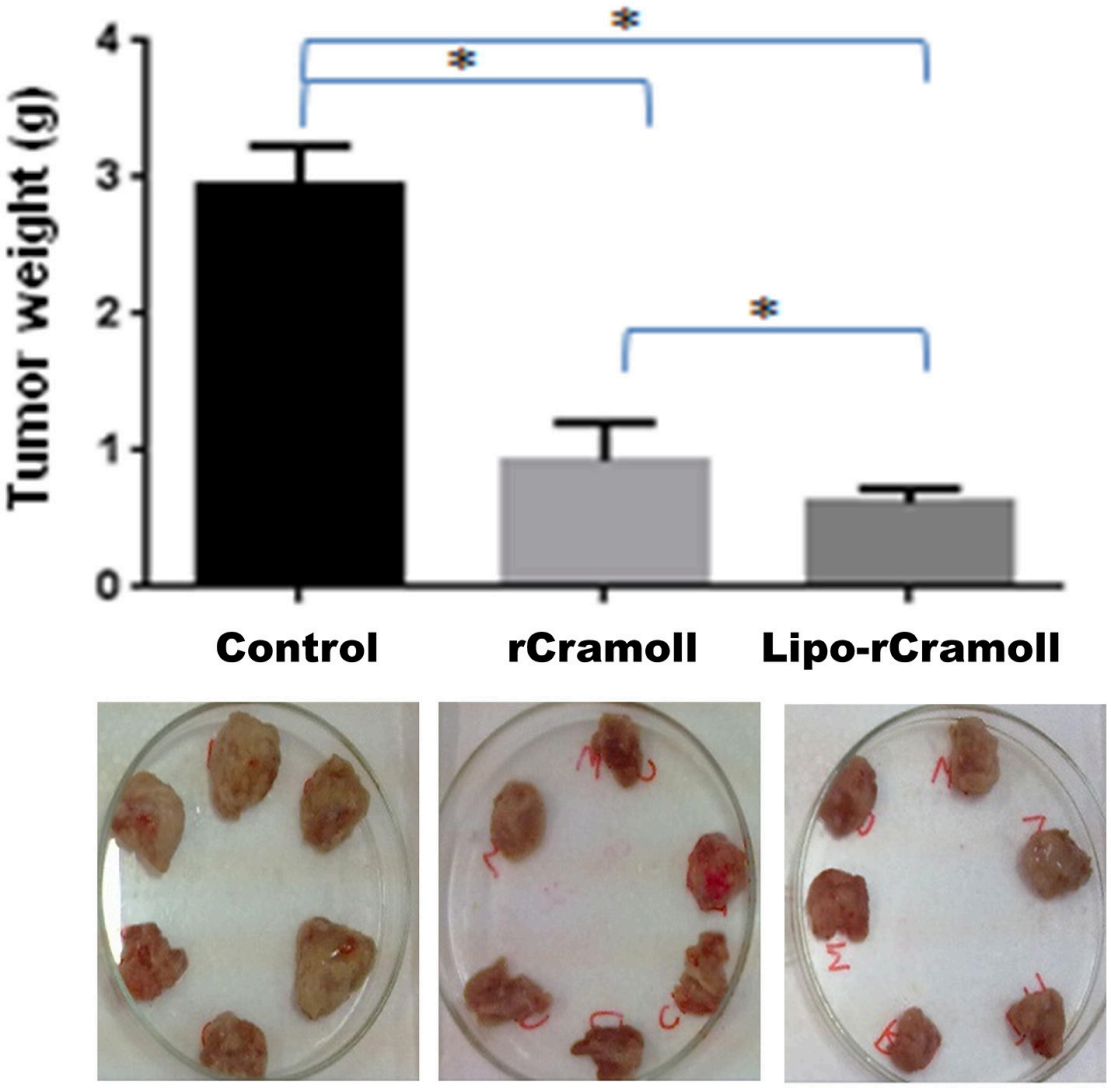
**Effects of rCramoll and rCramoll-loaded stealth liposomes (rCramoll-lipo) on weight of sarcoma 180 tumors**. ^*^(*p* < 0.05).

Figure 1 shows the antitumor activity of rCramoll and rCramoll-loaded stealth liposomes on mice transplanted with sarcoma 180 tumors. Both free and encapsulated rCramoll treatments resulted in high levels of *in vivo* tumor growth inhibition. rCramoll showed antitumor activity of 59% with a reduction of 0.82 ± 0.17 g in tumor mass. On the other hand, the encapsulation of rCramoll in stealth liposomes promoted a greater reduction in tumor mass (0.50 ± 0.10 g) resulting in 75% of growth inhibition. In other words, rCramoll-lipo was 16% more efficient than free lectin (*p* < 0.05).

### Histopathological analysis

**Figure 2:**
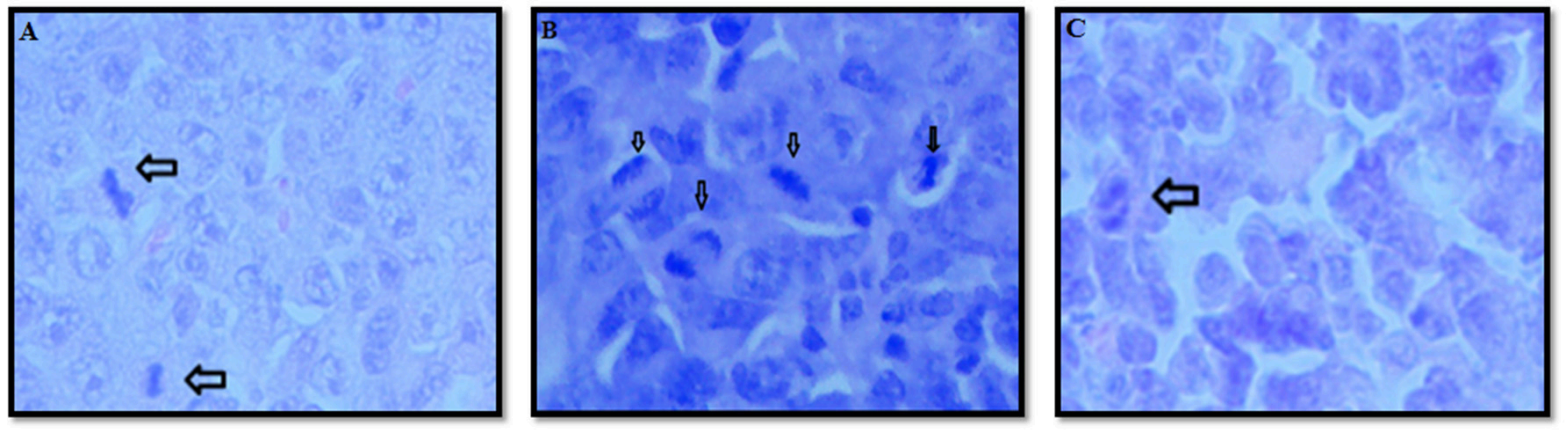
**Analysis of cell division in tumor tissue from untreated animals (A) and treated with rCramoll (B) and rCramoll-loaded stealth liposomes (C)**. Cell division (mitosis) are indicated by arrow.

**Figure 3:**
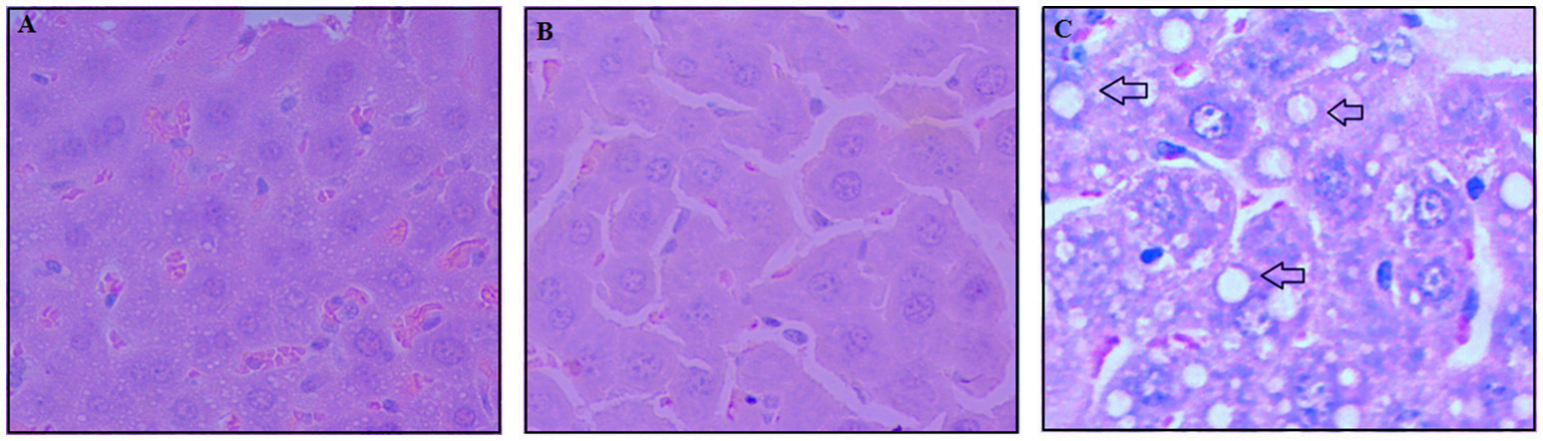
**Histopathological analysis of liver from untreated animals (A) and treated with rCramoll (B) and rCramoll-loaded stealth liposomes (C)**. Vacuolization areas are indicated by arrow.

Regardless of the group studied, the tumor invaded muscle, bone and fat tissue. Areas of necrosis and hemorrhage were also observed. The neoplastic cells were predominantly arranged in solid pattern, pleomorphic, large, with abundant cytoplasm and without defined borders. A significant inhibition of cell division (mitotic index) was observed in the animals treated with rCramoll-lipo (~5%, in relation to control group); otherwise, rCramoll promoted more cell proliferation (13.76%) in relation to untreated animals (*p* < 0.05; Figure 2). Histopathological analysis of selected organs of animals treated with rCramoll and rCramoll-lipo groups showed no changes in kidneys and spleen morphology. However, vacuolization areas were observed in hepatocytes from animals treated with encapsulated lectin, suggesting the presence of early steatosis and microabscess, both related to inflammatory process (Figure 3).

### Hematological and biochemistry analysis

Analysis of hematological parameters (Table 5) of control and treated animals revealed interesting alterations: All experimental groups showed significant changes in relation to healthy animals. rCramoll treated group exhibited an increase in leukocyte number (8.1 ± 3.0 10^3^/μL) when compared with both normal (3.2 ± 0.12 10^3^/μL) and untreated animals (11.4 ± 1.7 10^3^/μL). Surprisingly, the lectin encapsulation had a stronger effect on leukocyte number (17.1 ± 3.2 10^3^/μL), which represented an increment of 111.11%. Other cellular parameters (Ht, MCV, MCH, Hb, MCHC, WBC, RBC) were virtually unaltered by treatment.

**Table 5:** **Hematological and biochemistry analysis of blood samples of mice treated with rCramoll and rCramoll-lipo**.

**Parameters**	**Treatments**			
	**Healthy**	**Untreated (Saline)**	**rCramoll**	**rCramoll-lipo**
Hb (g/dL)	18.5 ± 0.05	12.6 ± 2.4^‡^	13.4 ± 1.3^‡^	10.3 ± 3.1^‡^, ^*^
Ht (%)	51.0 ± 0.44	39.1 ± 7.3^‡^	40.7 ± 3.8^‡^	31.3 ± 9.8^‡^
MCV (μm^3^)	52.8 ± 0.49	50 ± 1.7^‡^	47 ± 0.5^‡^	48 ± 1.4^‡^
MCH (pg)	17.8 ± 0.05	16 ± 0.5^‡^	15.6 ± 0.2^‡^	15.8 ± 0.3^‡^
MCHC (g/dL)	33.4 ± 0.57	32.4 ± 0.3^‡^	33.0 ± 0.2^‡^, ^*^	33.0 ± 0.4^‡^
RDW (%) (10^6^/μL)	10.4 ± 0.03	7.93 ± 1.6^‡^	8.6 ± 0.9^‡^	6.56 ± 2.1^‡^, ^*^
WBC (10^3^/μL)	3.2 ± 0.12	11.4 ± 1.7^‡^	8.1 ± 3.0^‡^	17.1 ± 3.2^‡^, ^*^
Glucose (mg/dL)	116.1 ± 10.4	178.3 ± 36.2	148.5 ± 26.0	177.7 ± 43.9
Urea (mg/dL)	27.67 ± 3.78	37.6 ± 15.5	35.5 ± 5.3	33.8 ± 3.3
AST (U/L)	79.43 ± 7.50	276 ± 19^‡^	183 ± 50^‡^, ^*^	217 ± 30^‡^, ^*^
ALT (U/L)	31.4 ± 2.1	20.4 ± 3.6	25.7 ± 18.5	9.4 ± 4.0^‡^, ^*^

‡ Significant differences in relation to control;

* *Significant differences in relation to control (saline) treated animals. Legend: Hemoglobin concentration (Hb), hematocrit (Ht), mean corpuscular volume (MCV), mean corpuscular hemoglobin (MCH), mean corpuscular hemoglobin concentration (MCHC), white blood cells (WBC), red blood cells (RBC), aspartate aminotransferase (AST), and alanine aminotransferase (ALT)*.

**Figure 4:**
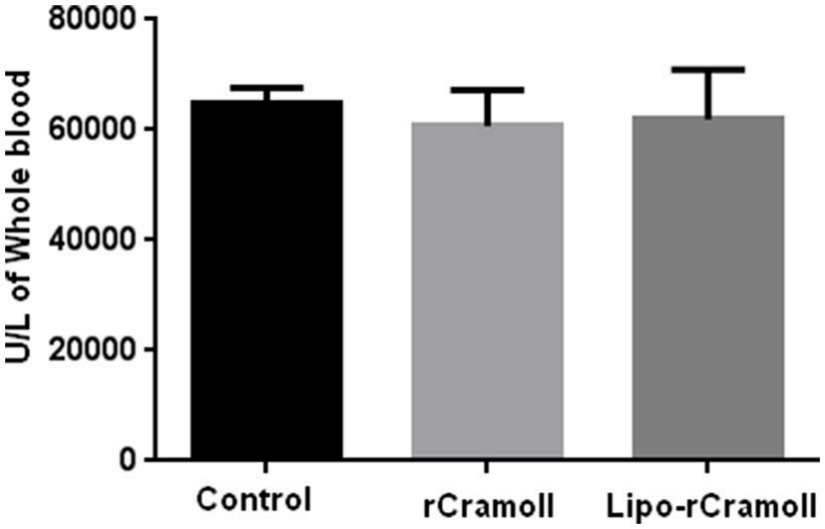
**Effects of rCramoll and rCramoll-lipo on serum levels of gluthathione peroxide**.

No significant changes in urea or glucose level were seen between any sarcoma 180 tumor-transplanted animals (treated or untreated) and healthy animals (*p* < 0.05); discarding possible kidney damage. On the other hand, the enzymatic activity of aspartate aminotransferase was significantly reduced in animals treated with free (183.5 ± 5 IU/L) or encapsulated lectin (217 ± 3 IU/L) when compared to untreated group (276 ± 19 IU/L). Regarding alanine aminotransferase activity, treatment-related effects were observed and their levels were not significantly different than the control group, except for encapsulated rCramoll treated group, which showed a significant reduction (9.4 ± 4.0 IU/L; Table 5). Furthermore, the levels of glutathione peroxidase were not altered by both treatments with rCramoll or its encapsulated form (Figure 4).

## Discussion

New molecular approaches using modified microorganism have been developed for production of recombinant proteins, allowing them to be used as research, therapeutic and diagnostic tools (Pina et al., 2013). Specifically, the heterologous expression of rCramoll and other bioactive lectins offer some advantages such as any seasonal interference or plant collection, production of high amounts of proteins with defined amino-acid sequences (excluding the presence of different isoforms) leading to more precise biomedical properties (Varejão et al., 2010; Oliveira et al., 2014). rCramoll was expressed using a sophisticated approach which resulted in a functional protein with several physicochemical properties (molecular mass, charge density, sugar specificity and tertiary structures) similar to its native counterpart. However, a little enhancement in stability to acidification, high temperatures and hydrostatic pressure were observed (Varejão et al., 2011).

A strong antitumor action has been reported for pCramoll (native protein) free and loaded in conventional liposomes (Andrade et al., 2004). These authors showed that the encapsulation of pCramoll improved its antitumor activity (30%) and decreased its tissue toxicity (particularly, in the liver and kidney). Taken together, these factors pointed to the need to evaluate the antitumor action of the new lectin rCramoll. For this purpose the nanoencapsulation of rCramoll into stealth liposome was employed to improve its therapeutic ratio as a result of EPR effect, which reduces drug concentrations in normal tissues and raises their concentrations in tumors (Sur et al., 2014). The EPR phenomenon has become the gold standard in the design of anticancer agents (Fang et al., 2011; Torchilin, 2011).

The first step was to evaluate the effects of encapsulation parameters in rCramoll functional stability by HA. The results showed that rCramoll was stable up to 2 freezing-thawing cycles, 250 s of ultrasound and 48 h of mechanical agitation. The results were similar to those found for pCramoll (Andrade et al., 2004). Then, an encapsulation ratio of 60% was obtained after 2 cycles of freezing-thawing, without altering rCramoll stability. The freeze-thaw technique is widely used to encapsulate hydrophilic molecules into liposomes, and it is appropriate to increase the internal volume of these vesicles (Sriwongsitanont and Ueno, 2011).

In this work, liposomes were formulated with 5% of PEG (polyethylene glycol) to ensure stealth nanosystems for *in vivo* application (Dos Santos et al., 2007). However, the addition of PEG can influence the encapsulation efficiency of hydrophilic macromolecules because of the presence of the PEG chains within the vesicles which may restrict the available of internal space for encapsulation (Nicholas et al., 2000), this may explain the reduction about 26% observed in rCramoll encapsulation efficiency in stealth liposomes, compared to Andrade et al. (2004) for pCramoll in conventional liposomes.

Proteins are macromolecules bigger than conventional drugs, and several parameters influence the performance of their encapsulation, including the concentration of lipids and size (diameter) of the liposomes (Hwang et al., 2012). The increase in lipid concentration results in a greater inner volume; therefore, in this study liposomes were prepared using 80 mM. Additionally, the diameter of vesicles was 168 ± 0.7 nm, which is considered within the optimal range (<200 nm) for antitumor therapy due to the capacity to accumulate passively in sites with increased vascular permeability (Allen and Cullis, 2013). The PDI of the liposomal formulation was 0.265, this parameter evaluates the size distribution having a value between 0 and 1 (the value 0 indicates the proximity of monodisperse particles; Jain et al., 2012). After the establishment of the best parameters for the formulation of rCramoll-lipo, the antitumor potential of this nanosystem and free lectin were evaluated. They were intraperitoneally administered. The administration of substances into the peritoneal cavity (a parenteral route) is common in laboratory rodents for which intravenous access can be used to safely inject large volumes of fluid. Once in blood circulation, the agent (compound or a formulation) may be conducted for different areas in the body, including for the subcutaneous tumor area (which is highly vascularized; Turner et al., 2011). This route has been widely used for *in vivo* evaluations of antitumor activity (Goh et al., 2015; Ferreira et al., 2016; Silveira et al., 2016).

Our results revealed that the tumor inhibition induced by lipo-rCramoll was 16% higher than free rCramoll. Additionally, antitumoral activity of free rCramoll was more potent when compared with the previous results reported by Andrade et al. (2004) for free pCramoll. On the other hand, even with an encapsulation efficiency 20% lower than pCramoll-lipo, similar results were found between rCramoll-lipo and pCramoll-lipo antitumor activities, advocating thereby for a stronger action of rCramoll-lipo (Andrade et al., 2004). The improved activity of drugs in stealth liposomes has been shown for other antitumor agents, for example, gemcitabine hydrochloride showed improved pharmacokinetics and residence time, and reduced blood toxicity (Pitrubhakta et al., 2012).

Another important parameter to an antitumor agent is the mitotic index which revealed that the encapsulation of rCramoll was able to prevent the induction of tumor proliferation (*p* < 0.05), while free rCramoll induced cell division. The mitogenic action of free pCramoll has been related to its well-known immunomodulatory potential (Nascimento da Silva et al., 2014). On the other hand, as nanosystem (stealth liposomes) provided drug delivery in a controlled manner, rCramoll-lipo seems to be more effective on acting in the recruitment of immune cells culminating in tumor reduction. In fact, animals treated with encapsulated protein exhibited an increase in the leukocyte number when compared with others groups. Inflammation mediated by leukocytes is an essential immune response to perturbed tissue homeostasis and plays decisive roles at different stages of tumor growth, affecting the responses to therapy (positively or negatively; Mantovani et al., 2008). For example, both chemotherapy and radiation kill cancer cells mostly through necrosis, a proinflammatory form of cell death, while several anti-inflammatory drugs have been found to reduce tumor incidence (Grivennikov et al., 2010). Previous studies have indicated that pCramoll is an immunomodulatory agent able to induce Th1 and Th17 responses (Nascimento da Silva et al., 2014). Recently, the immunomodulatory potential of rCramoll has been also demonstrated using mouse peritoneal exudate cells (mPECs). rCramoll treatment significantly enhanced the production of proinflammatory cytokines by PECs (IL-1β, IL-6, IFN-γ, and TNF-α) and nitric oxide (NO; da Silva et al., 2015). Therefore, at this point, a crucial difference between the anti-tumor action of free and encapsulated rCramoll was found along with their effects on immune cells.

Concerning the effects of both treatments on kidneys, no significant alterations were observed in the urea level and kidneys morphology. Likewise, the levels of AST were unaltered among the different experimental groups. However, a significant decrease in ALT levels was observed in the group treated with the encapsulated lectin. The histopathological analysis showed the presence of inflammatory infiltrates and areas of microsteatosis in the liver of animals treated with the encapsulated rCramoll, however, interstitial fibrosis, necrosis, or parenchymal involvement were not observed in this group. In fact, in a study of biodistribution, large quantities of pCramoll were found in the liver of animals (Patricio et al., 2011). Thus, without the occurrence of more serious damage, the liver is capable of promoting its regeneration (Scheuer, 2003).

Finally, any treatment-related effects were observed in the levels of glutathione peroxidase. This enzyme is an important component of the antioxidant defense system, and as tumor cells generally produce much more reactive oxygen than normal cells, the expression of protective enzymes may be compromised in some cancers (Falck et al., 2010). However, other studies also showed that, in some types of tumor cells, a decreased expression of glutathione peroxidase can enhance tumor suppressor effect of manganese enzyme superoxide dismutase (MnSOD), which is another important antioxidant enzyme (Liu et al., 2004). It is worth noting that the induction of reactive oxygen species (ROS) by pCramoll has been demonstrated in several murine models (Nascimento da Silva et al., 2014), and rCramoll is also able to increase the *in vitro* ROS production by mPECs (da Silva et al., 2015). Both lectins are able to induce cell proliferation even in cells exposed to oxidative stress (Nascimento da Silva et al., 2015). Given the action of both free rCramoll and rCramoll-lipo on leukocytes proliferation, ROS may be involved in the antitumoral action of lectin, and they would act oxidizing glutathione and consequently inhibiting the action of GPX. Induction of free radicals is related to the activity of many antitumor drugs (Lampiasi et al., 2012; Chung et al., 2013). Other tests should also be performed in order to investigate involvement of ROS in anti-tumor capacity of rCramoll.

## Conclusion

In conclusion, the encapsulation of rCramoll in stealth liposomes improves its antitumor activity without substantial toxicity. This approach was more successful than the previous results reported for pCramoll loaded into conventional liposomes. Additionally, the effects of lectin in the mitotic index were also prevented by encapsulation. The enhancement on antitumoral action seems to be related to increment of leukocytes account during the encapsulated rCramoll treatment. Therefore, further investigations are required to elucidate the mechanism(s) of the rCramoll antitumor effect.

## Author contributions

CC, LS, LC, NSS, and MTSC conceived the study, and participated in its design and coordination. CC, LS, and NV performed the rCramoll expression, purification and hemagglutination assays. CC, FJFA, and MF participated in liposome preparation and in vivo studies. CC, FCAA, NPS, MFSC performed the histopathological analysis. CC, LS, RM, NSS, MTSC: Analyzed the data and drafted the manuscript. All authors read and approved the final manuscript.

### Conflict of interest statement

The authors declare that the research was conducted in the absence of any commercial or financial relationships that could be construed as a potential conflict of interest.

## References

[B1] Allen T. M. Cullis P. R. (2013). Liposomal drug delivery systems: from concept to clinical applications. Adv. Drug Deliv. Rev. 65, 36–48. 10.1016/j.addr.2012.09.037 23036225

[B2] Andrade C. A. S. Correia M. T. S. Coelho L. C. B. B. Nascimento S. C. Santos-Magalhães N. S. (2004). Antitumor activity of *Cratylia mollis* lectin encapsulated into liposomes. Int. J. Pharm. 278, 435–445. 10.1016/j.ijpharm.2004.03.028 15196647

[B3] Chung K. S. Han G. Kim B. K. Kim H. M. Yang J. S. Ahn J.. (2013). A novel antitumor piperazine alkyl compound causes apoptosis by inducing RhoB expression via ROS-mediated c-Abl/p38 MAPK signaling. Cancer Chemother. Pharm. 72, 1315–1324. 10.1007/s00280-013-2310-y 24121479

[B4] Cleland J. L. Daugherty A. Mrsny R. (2001). Emerging protein delivery methods. Curr. Opin. Biotechnol. 12, 212–219. 10.1016/S0958-1669(00)00202-0 11287240

[B5] Correia M. T. S. Coelho L. C. B. B. (1995). Purification of glucose/manose specific lectin, isoform 1, from seeds of *Cratylia mollis* Mart. (Camaratu bean). Appl. Biochem. Biotechnol. 55, 261–273. 10.1007/BF02786865 8579345

[B6] Danhier F. Feron O. Préat V. (2010). To exploit the tumor microenvironment: passive and active tumor targeting of nanocarriers for anti-cancer drug delivery. J. Control Release 148, 135–146. 10.1016/j.jconrel.2010.08.027 20797419

[B7] da Silva L. C. N. Alves N. M. P. Castro M. C. A. B. Pereira V. R. A. Paz N. V. N. Coelho L. C. B. B.. (2015). Immunomodulatory effects of pCramoll and rCramoll on peritoneal exudate cells (PECs) infected and non-infected with *Staphylococcus aureus*. Int. J. Biol. Macromol. 72, 848–854. 10.1016/j.ijbiomac.2014.09.045 25305338

[B8] Da Silva L. C. N. Correia M. T. S. (2014). Plant Lectins and Toll-like receptor: implications in therapy for microbial infections. Front. Microbiol. 5:20. 10.3389/fmicb.2014.00020 24550893 PMC3909824

[B9] Dos Santos N. Allen C. Doppen A. M. Anantha M. Cox K. Gallagher R. C.. (2007). Influence of poly(ethylene glycol) grafting density and polymer length on liposomes: relating plasma circulation lifetimes to protein binding. Biochim. Biophys. Acta 1768, 1367–1377. 10.1016/j.bbamem.2006.12.013 17400180

[B10] Falck E. Karlsson S. Carlsson J. Helenius G. Karlsson M. A. T. S. Klinga-Levan K. (2010). Loss of glutathione peroxidase 3 expression is correlated with epigenetic mechanisms in endometrial adenocarcinoma. Cancer Cell Int. 10, 1–9. 10.1186/1475-2867-10-46 21106063 PMC3014921

[B11] Fang J. Nakamura H. Maeda H. (2011). The EPR effect: unique features of tumor blood vessels for drug delivery, factors involved, and limitations and augmentation of the effect. *Adv*. Drug Deliv. Rev. 63, 136–151. 10.1016/j.addr.2010.04.009 20441782

[B12] Ferreira P. M. P. Bezerra D. P. Nascimento Silva J. Costa M. P. Oliveira Ferreira J. R. Alencar N. M. N.. (2016). Preclinical anticancer effectiveness of a fraction from *Casearia sylvestris* and its component Casearin X: *in vivo* and *ex vivo* methods and microscopy examinations. J. Ethnopharmacol. 186, 270–279. 10.1016/j.jep.2016.04.011 27067367

[B13] Fu L. L. Zhou C. C. Yao S. Yu J. Y. Liu B. O. Bao J. K. (2011). Plant lectins: targeting programmed cell death pathways as antitumor agents. *Int*. J. Biochem. Cell Biol. 43, 1442–1449. 10.1016/j.biocel.2011.07.004 21798364

[B14] Ghazarian H. Idoni B. Oppenheimer S. B. (2011). A glycobiology review: carbohydrates, lectins and implications in cancer therapeutics. Acta Histochem. 113, 236–247. 10.1016/j.acthis.2010.02.004 20199800 PMC3027850

[B15] Goh A. R. Shin S. P. Jung N. R. Ryu C. H. Eom H. S. Lee J. H.. (2015). Low-dose cisplatin converts the tumor microenvironment into a permissive state for HSVtk-induced antitumor immunity in HPV16-related tonsillar carcinoma. Cancer Lett. 356, 743–750. 10.1016/j.canlet.2014.10.022 25449436

[B16] Grivennikov S. I. Greten F. R. Karin M. (2010). Immunity, inflammation, and cancer. Cell 140, 883–899. 10.1016/j.cell.2010.01.025 20303878 PMC2866629

[B17] Hwang S. Y. Kim H. K. Choo J. G. Seong H. Hien T. B. D. Lee E. K. (2012). Effects of operating parameters on the efficiency of liposomal encapsulation of Enzymes. Colloids Surf. B Biointerfaces 94, 296–303. 10.1016/j.colsurfb.2012.02.008 22398367

[B18] Jain S. Kumar D. Swarnakar N. K. Thanki K. (2012). Polyelectrolyte stabilized multilayered liposomes for oral delivery of paclitaxel. Biomaterials 33, 6758–6768. 10.1016/j.biomaterials.2012.05.026 22748771

[B19] Lampiasi N. Azzolina A. Umezawa K. Montalto G. Mccubrey J. A. Cervello M. (2012). The novel NF-κB inhibitor DHMEQ synergizes with celecoxib to exert antitumor effects on human liver cancer cells by a ROS-dependent mechanism. Cancer Lett. 322, 35–44. 10.1016/j.canlet.2012.02.008 22343223

[B20] Liu B. Bian H. J. Bao J. K. (2010). Plant lectins: potential antineoplastic drugs from bench to clinic. Cancer Lett. 287, 1–12. 10.1016/j.canlet.2009.05.013 19487073

[B21] Liu G. Zhou W. Wang L. I. Park S. Miller D. P. Xu L. L.. (2004). MPO and SOD2 polymorphisms, gender, and the risk of non-small cell lung carcinoma. Cancer Lett. 214, 69–79. 10.1016/j.canlet.2004.06.027 15331175

[B22] Mantovani A. Allavena P. Sica A. Balkwill F. (2008). Cancer-related inflammation. Nature 454, 436–444. 10.1038/nature07205 18650914

[B23] Nascimento da Silva L. C. Alves N. M. P. de Castro M. C. A. B. Higino T. M. M. da Cunha C. R. A. Pereira V. R. A. (2015). pCramoll and rCramoll as new preventive agents against the oxidative dysfunction induced by hydrogen peroxide. Oxid. Med. Cell. Longev. 2015:520872. 10.1155/2015/520872 26576224 PMC4632182

[B24] Nascimento da Silva L. C. Bezerra Filho C. M. Paula R. A. Coelho L. C. B. B. Silva M. V. Correia M. T. S. (2014). *Cratylia mollis* lectin: a versatile tool for biomedical studies. Curr. Bioact. Comp. 10, 44–54. 10.2174/157340721001140725000701

[B25] Nicholas A. R. Scott M. J. Kennedy N. I. Jones M. N. (2000). Effect of grafted polyeth-ylene glycol (PEG) on the size, encapsulation efficiency and permeability of vesicles. Biochim. Biophys. Acta 1463, 167–178. 10.1016/S0005-2736(99)00192-3 10631306

[B26] Oliveira C. Teixeira J. A. Domingues L. (2014). Recombinant production of plant lectins in microbial systems for biomedical application–the frutalin case study. Front Microbiol. 5:390. 10.3389/fpls.2014.00390 25152749 PMC4126444

[B27] Patricio B. F. C. Lima-Ribeiro M. H. M. Correia M. T. S. Carneiro-Leão A. M. A. Albernaz M. S. Barboza T.. (2011). Radiolabeling of Cramoll 1,4: evaluation of the Biodistribution. Int. J. Pept. 2011, 1–3. 10.1155/2011/945397 21760823 PMC3133851

[B28] Pick U. (1981). Liposomes with a large trapping capacity prepared by freezing and thawing of sonicated phospholipid mixture. *Arch*. Biochem. Biophys. 212, 186–194. 10.1016/0003-9861(81)90358-1 7197900

[B29] Pina A. S. Lowe C. R. Roque C. A. (2013). Challenges and opportunities in the purification of recombinant tagged proteins. *Biotechnol*. Adv. 32, 366–381. 10.1016/j.biotechadv.2013.12.001 24334194 PMC7125906

[B30] Pitrubhakta A. B. Shinde A. J. Jadhav N. R. (2012). Design, development and characterization of PEGylated liposomes of gemcitabine hydrochloride. Der. Pharm. Lett. 4, 314–329.

[B31] Ryan R. J. H. Bernstein B. E. (2012). Genetic events that shape the cancer epigenome. Science 336, 1513–1514. 10.1126/science.1223730 22723401

[B32] Scheuer P. J. (2003). Liver biopsy size matters in chronic hepatitis: bigger is better. Hepatology 38, 1356–1358. 10.1016/j.hep.2003.10.010 14647044

[B33] Silveira C. P. Apolinário L. M. Favaro W. J. Paula A. J. Duran N. (2016). Doxorubicin-functionalized silica nanoparticles incorporated into a thermoreversible hydrogel and intraperitoneally administered result in high prostate antitumor activity and reduced cardiotoxicity of doxorubicin. ACS Biomater. Sci. Eng. 2, 1190–1199. 10.1021/acsbiomaterials.6b00241 33465877

[B34] Sriwongsitanont S. Ueno M. (2011). Effect of freeze-thawing process on the size and lamellarity of peg-lipid liposomes. Open Colloid Sci. J. 4, 1–6. 10.2174/1876530001104010001

[B35] Sur S. Fries A. C. Kinzler K. W. Zhou S. Vogelstein B. (2014). Remote loading of preencapsulated drugs into stealth liposomes. Proc. Natl. Acad. Sci. U.S.A. 111, 2283–2288. 10.1073/pnas.1324135111 24474802 PMC3926059

[B36] Torchilin V. (2011). Tumor delivery of macromolecular drugs based on the EPR effect. *Adv. Drug Deliv*. Rev. 63, 131–135. 10.1016/j.addr.2010.03.011 20304019

[B37] Turner P. V. Pekow C. Vasbinder M. A. Brabb T. (2011). Administration of substances to laboratory animals: equipment considerations, vehicle selection, and solute preparation. J. Am. Assoc. Lab. Anim. Sci. 50, 614–627. 22330706 PMC3189663

[B38] Varejão N. Almeida M. S. De Cicco N. N. T. Atella G. C. Coelho L. C. B. B. Correia M. T. S.. (2010). Heterologous expression and purification of a biologically active legume lectin from *Cratylia mollis* seeds (CRAMOLL 1). Biochim. Biophys. Acta 1804, 1917–1924. 10.1016/j.bbapap.2010.06.004 20538076

[B39] Varejão N. Correia M. T. S. Foguel D. (2011). Characterization of the unfolding process of the tetrameric and dimeric forms of *Cratylia mollis* seed lectin (CRAMOLL 1): effects of natural fragmentation on protein stability. Biochemistry 50, 7330–7340. 10.1021/bi200320x 21790141

[B40] Walde P. Ichikawa S. (2001). Enzymes inside lipid vesicles: preparation, reactivity and applications. Biomol Eng. 18, 143–177. 10.1016/S1389-0344(01)00088-0 11576871

